# The impact of public access defibrillation on outcomes for children experiencing out-of-hospital cardiac arrest: a 25-year retrospective study

**DOI:** 10.1016/j.resplu.2026.101278

**Published:** 2026-02-18

**Authors:** Nora Abu-Obeid, Zainab Alqudah, Ashanti Dantanarayana, Ziad Nehme

**Affiliations:** a Department of Allied Medical Sciences, Jordan University of Science and Technology, Irbid, Jordan; b Department of Paramedicine, Monash University, Frankston, Victoria, Australia; c Centre for Research and Evaluation, Ambulance Victoria, Blackburn North, Victoria, Australia; d School of Public Health and Preventive Medicine, Monash University, Victoria, Australia

**Keywords:** Public access defibrillation, Paediatrics, OHCA, Cardiac arrest, AED

## Abstract

**Aim:**

To evaluate the impact of public access defibrillation (PAD) on outcomes for children experiencing out-of-hospital cardiac arrest (OHCA).

**Methods:**

This retrospective cohort study analysed paediatric (age <18 years), non-traumatic OHCA cases with an initial shockable rhythm from the Victorian Ambulance Cardiac Arrest Registry between 1 January 2000 and 31 December 2024. Cases witnessed to arrest by emergency medical services (EMS) were excluded. Cases were categorised based on the initial shock provider, either PAD or EMS. The association between PAD and patient outcomes was assessed in multivariable logistic regression models.

**Results:**

The study identified 140 shockable OHCA cases meeting the eligibility criteria, of which 23 (16.4%) received their first defibrillation via PAD. PAD use increased over the study period, from 0% in 2000–04 to 35.7% in 2020–24 across all locations and from 0% to 66.7% in public locations (*p* trend <0.001 for both). PAD use was significantly associated with improved unadjusted odds of ROSC (OR: 6.33; 95% CI: 1.42–28.30; *p* = 0.016), event survival (OR: 3.80; 95% CI: 1.22–11.86; *p* = 0.022), and survival to hospital discharge (OR: 5.11; 95% CI: 1.63–15.99; *p* = 0.005). However, these associations were not statistically significant after adjusting for confounders including the temporal trend. Survival to hospital discharge improved significantly over time (OR: 10.44; 95% CI: 2.26–48.15 for 2020–24 vs. 2000–04).

**Conclusion:**

PAD in children increased significantly in our region over 25 years and was associated with a marked reduction in the time to first defibrillation.

## Introduction

Out of hospital cardiac arrest in children is a rare but devastating event, with an estimated incidence of 4.9–6.7 events per 100,000 persons.^1^ Survival rates remain poor, with estimates ranging between 12.1% and 12.9%.^2^ Survival depends on the effectiveness of the “chain of survival” which consists of early recognition and activation of emergency medical services (EMS), early bystander cardiopulmonary resuscitation (CPR), early defibrillation and advanced life support (ALS).^3^, ^4^ In cases of OHCA with shockable rhythms, studies have reported survival rates as high as 69% when defibrillation is performed within the first few minutes after collapse.^5^, ^6^, ^7^ As a result, Public Access Defibrillation (PAD) programs have been established in many communities to reduce the time to first shock.^8^, ^9^

Although PAD effectiveness is well established in adults, less is known about its impact in children.^10^, ^11^ A recent systematic review by the International Liaison Committee on Resuscitation found that the application of PAD was associated with improved outcomes in children aged 1–18 years, including higher rates of survival to hospital discharge and favourable neurological outcomes (CPC 1–2).^12^ However, the review was limited by serious risk of bias and heterogeneity of the included studies, which precluded meta-analysis. The data presented in the review were also crude comparisons of the relative risk of patient outcomes comparing those with and without PAD application/shock, which may also introduce confounding bias.

Given these limitations, there is a need to better understand the role of PAD in children experiencing OHCA. To address this, we sought to examine temporal trends in the use of PAD for paediatric OHCA in Victoria, Australia, and explore its impact on survival outcomes.

## Methods

### Study design

We performed a retrospective analysis of paediatric (aged <18 years) nontraumatic OHCA cases with an initial shockable rhythm occurring between 1st January 2000 and 31st December 2024. Patients witnessed to arrest by EMS personnel, those with initial non-shockable rhythms and those with traumatic aetiology of arrest were excluded. The study, including the collection and use of data from the Victorian Ambulance Cardiac Arrest Registry (VACAR), was approved by the Monash University Human Research Ethics Committee (Project ID 45498) and Ambulance Victoria’s Research Committee.

### Setting

The study was conducted in the state of Victoria, Australia, which has a population of approximately 6.8 million people and is served by a single, state-wide EMS, Ambulance Victoria. In 2010 (the study midpoint), the population of children under 18 years in Victoria was approximately 1.22 million, representing 21.9% of the state’s total population, while those under 12 years were estimated at around 417,750, accounting for 7.5% of the total population.^13^ Ambulance Victoria operates a two-tiered EMS across the state, staffed by advanced life support (ALS) and Mobile Intensive Care Ambulance (MICA) Paramedics, who are equipped to deliver advanced airway management, cardiac life support, and pharmacological interventions at the scene of a cardiac arrest.^14^ Since 2018, the state has utilised the GoodSAM smartphone application to alert registered responders to nearby cardiac arrests and registered local PADs.^15^ While Ambulance Victoria maintains a public facing AED register it is not integrated into the emergency dispatch system. A formal PAD program was established in Victoria in 2002,^16^ which ensures the maintenance of a select number of AEDs at public locations including railway stations, airports and major tourist venues. In addition to the AV PAD program, AEDs are also available in the Melbourne casino, schools, workplaces, and shopping and recreational areas. There is no legislation mandating the application of PADs in public places in Victoria.

### Source of data

Data were obtained from the Victorian Ambulance Cardiac Arrest Registry (VACAR), which collects information on all EMS attended OHCA cases across the state, offering a consistent and comprehensive dataset. The registry has been collecting data since 1999 on all patients in Victoria who experience an OHCA and receive an attendance from paramedics. Further information on the registry’s methodology has been described elsewhere.^17^ These data were sourced from electronic patient care records and operational databases and were collected in accordance with the internationally recognised Utstein criteria.^18^

### Definitions

The definitions applied in this study were based on the internationally recognised Utstein reporting guidelines.^18^ For the purpose of analysis, bystander CPR was defined as any chest compression attempt made prior to EMS arrival, regardless of whether rescue breaths were provided. Event survival was defined as a palpable pulse upon arrival at the emergency department, as documented in the patient care records. Survival to hospital discharge referred to the patient’s release from acute hospital care. EMS response time was defined as the time measured from emergency call to arrival on scene. For the purposes of this study, 'shocked by EMS' was defined as an initial shock delivered by either paramedics and/or dispatched first responders (firefighters or community volunteers). In contrast, 'shocked by PAD' referred to any initial shock administered by a bystander using a public access defibrillator. The latter includes shocks delivered by bystanders alerted through smartphone technology after 2018 (i.e. GoodSAM).

### Study outcomes

The primary outcome was survival to hospital discharge. Secondary outcomes included event survival and prehospital ROSC.

### Data analysis

All statistical analyses were conducted using Stata version 19 (StataCorp, College Station, TX, USA). Baseline characteristics of the study population were summarised using descriptive statistics and compared across initial shock provider (PAD vs EMS). We presented categorical variables as frequencies and percentages, while continuous variables were presented depending on the data distribution, either as means with standard deviations or medians with interquartile ranges. We used chi-square test to compare categorical variables and the Mann-Whitney *U* test or the Kruskal–Wallis test for continuous variables as appropriate.

Long-term trends in the rate of first shocks by PAD across 5-year intervals (e.g. 2000–04, 2005–2009, 2010–2014, 2015–2019, 2020–2024) were assessed using Cochran-Armitage test for trend for all arrests and arrests in public locations. To assess the association between first shock by PAD and the study outcomes, we performed multivariable logistic regression analyses. In the multivariable models we adjusted for age and sex, and other factors that might have an influence on PAD use, including witness status, bystander CPR, presumed cardiac, location of arrest, urban setting, and year of arrest group. We did not adjust for time to first shock in the primary models, as this would attenuate the effect of shock provider on study outcomes. Instead, for a sensitivity analysis, we repeated the earlier models but also included the time to first shock (e.g. time from emergency call to first shock time). For each outcome we calculated the adjusted odds ratios (AORs) with 95% confidence intervals (CIs). A *p*-value of <0.05 was considered statistically significant.

## Results

### Sample population

**Fig. 1:**
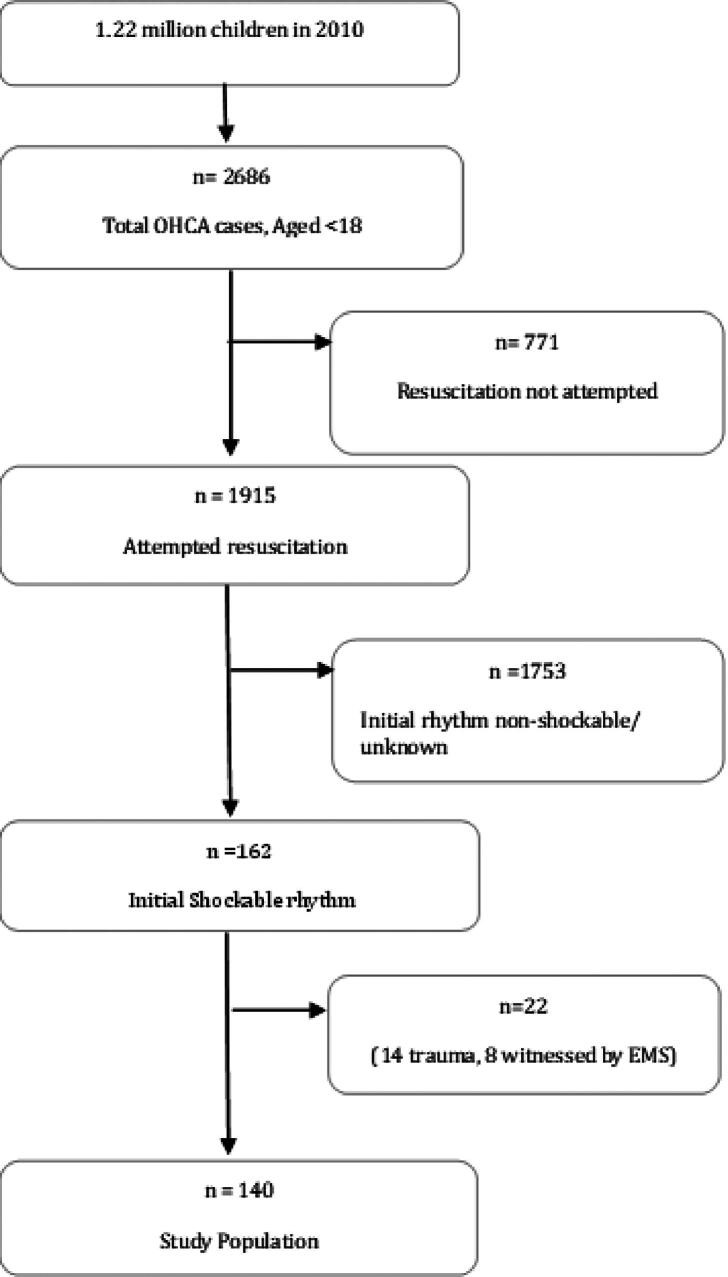
**Study flowchart on the selection of the study population in Victoria, Australia between January 1, 2000 and December 31, 2024**.

The patient selection flow chart is shown in Fig. 1. Between 2000 and 2024, a total of 2686 OHCA cases involving patients aged <18 years were attended by EMS in Victoria, Australia. Of these, 140 cases received an attempted resuscitation and were identified as non-traumatic arrests presenting with an initial shockable rhythm. Among these 140 cases, 117 patients (83.6%) received their first shock from EMS, while 23 patients (16.4%) were initially shocked by a PAD.

### Patient characteristics

Baseline characteristics stratified by shock provider are shown in Table 1. Compared to those initially shocked by EMS, patients initially shocked by PAD were more likely to arrest in public locations (91.3% vs. 41.9%; *p* < 0.001) and receive bystander CPR (95.7% vs. 74.4%; *p* = 0.025). In addition, the median time from emergency call to first shock was significantly shorter in cases first shocked by PAD (5.1 min [IQR: 3.5–10.0]) compared to EMS (11.0 min [IQR: 8.6–13.0]; *p* < 0.001).

**Table 1:** Characteristics of OHCA cases according to shock provider.

	**Overall** ***n* = 140**	**Shocked by PAD** ***n* = 23**	**Shocked by EMS** ***n* = 117**	***P*-values**	**Missing *n* (%)**
Age in years, median (IQR)	13 (6–16)	13 (11–15)	13 (5–16)	0.468	0
**Age groups, *n* (%)**					
*Infants*	17 (12.1%)	0	17 (14.5%)	0.145	0
*Children*	35 (25%)	7 (30.4%)	28 (23.9%)	0.145	0
*Adolescents*	88 (62.9%)	16 (69.6%)	75 (61.6%)	0.145	0
Male sex, *n* (%)	94 (67.1%)	17 (73.9%)	77 (65.8%)	0.450	0
**Arrest location, *n* (%)**					
*Private residence*	66 (47.1%)	<5	65 (55.6%)	<0.001	0
*Public location*	70 (50.0%)	21 (91.3%)	49 (41.9%)	<0.001	0
*Other*	<5	<5	<5	0.639	0
Presumed cardiac aetiology, *n* (%)	110 (78.6%)	21 (91.3%)	89 (76.1%)	0.104	0
Metropolitan region, *n* (%)	97 (69.3%)	18 (78.3%)	79 (67.5%)	0.307	0
Bystander witnessed, *n* (%)	108 (77.1%)	20 (87.0%)	88 (75.2%)	0.131	2 (1.4%)
Bystander CPR, *n* (%)	109 (77.9%)	22 (95.7%)	87 (74.4%)	0.025	0
Treatment by smartphone alerted community volunteer	8 (5.7%)	<5	6 (5.1%)	0.50	0
**Scene outcome, *n* (%)**					
*Efforts ceased at scene*	22 (15.7%)	<5	20 (17.1%)	0.312	0
*Transport with CPR ongoing*	34 (24.3%)	<5	32 (27.4%)	0.056	0
*Transport with ROSC*	84 (60.0%)	19 (82.6%)	65 (55.6%)	0.015	0
Call to EMS arrival time, median (IQR)	7.4 (6.0–9.9)	8.4 (6.4–10.6)	7.1 (6.0–9.9)	0.215	2 (1.4%)
Call to first shock time, median (IQR)	10.1 (7.8–13.0)	5.1 (3.5–10.0)	11.0 (8.6–13.0)	<0.001	7 (5.0%)
**Year of arrest, *n* (%)**					
*2000*–*2004*	32 (22.9%)	0	32 (27.4%)	<0.001	0
*2005*–*2009*	29 (20.7%)	<5	28 (23.9%)		0
*2010*–*2014*	15 (10.7%)	<5	13 (11.1%)		0
*2015*–*2019*	36 (25.7%)	10 (43.5%)	26 (22.2%)		0
*2020*–*2024*	28 (20.0%)	10 (43.5%)	18 (15.4%)		0

### Trends in shock provider

**Fig. 2:**
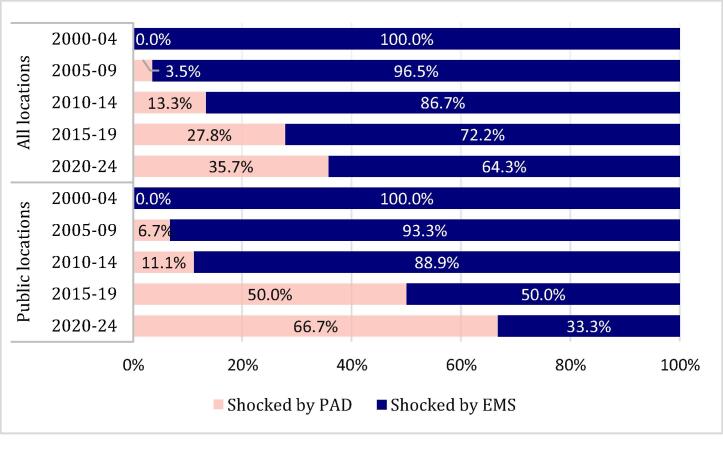
**Temporal trends in PAD use by location type (public vs all locations)**.

Fig. 2 displays the temporal trend in first shock provider between 2000 and 2024, across all locations and public locations. Across all locations, the rate of first shocks by PAD increased from 0% in 2000–2004 to 35.7% in 2020–24 (*p* for trend <0.001). Among cases in public locations, the rate of first shocks by PAD increased from 0% in 2000–2004 to 66.7% in 2020–24 (*p* for trend <0.001).

### Unadjusted outcomes

**Fig. 3:**
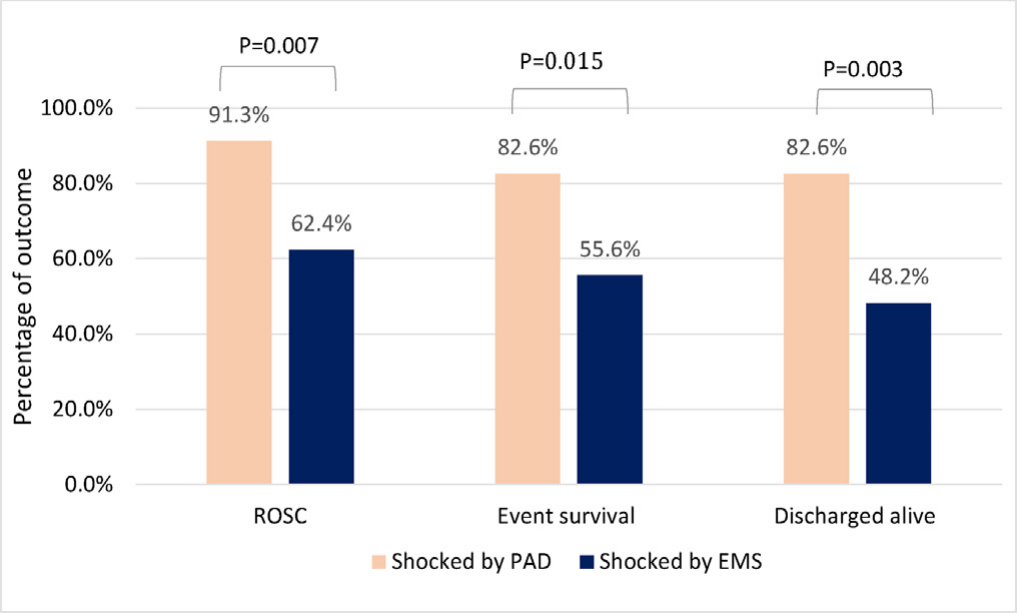
**Unadjusted rates of patient outcomes in paediatric OHCA cases, stratified by shock provider**.

Across the entire sample, the rate of prehospital ROSC was 67.1% (*n* = 94), event survival was 60.0% (*n* = 84), and survival to hospital discharge was 54.1% (*n* = 72). Fig. 3 compares unadjusted outcomes between patients initially shocked by PAD and those initially shocked by EMS. Patients in the PAD group had significantly higher rates of ROSC, event survival and survival to hospital discharge. In unadjusted logistic regression models, cases shocked first by PAD were associated with increased odds of survival to hospital discharge (OR: 5.11; 95% CI: 1.63–15.99; *p* = 0.005), event survival (OR: 3.80; 95% CI: 1.22–11.86; *p* = 0.022), and prehospital ROSC (OR: 6.33; 95% CI: 1.42–28.30; *p* = 0.016) compared to cases shocked first by EMS.

### Adjusted outcomes

Table 2 shows the results of the multivariable models of study outcomes. After adjustment for confounders, excluding time to first shock, cases shocked first by PAD were not associated with improved survival to hospital discharge, event survival or prehospital ROSC. The main factors driving improvement in survival to hospital discharge included bystander witness (OR: 3.76; 95% CI: 1.19–11.92; *P* = 0.024) and the year of arrest. There was a large temporal increase in the odds of survival that was independent of other arrest characteristics. Compared to the reference period (2000–2004), survival to hospital discharge was significantly higher in 2005–2009 (OR: 5.18; 95% CI: 1.35–19.96; *P* = 0.017), 2010–2014 (OR: 6.42; 95% CI: 1.26–32.76; *P* = 0.025), 2015–2019 (OR: 10.79; 95% CI: 2.72–42.78; *P* = 0.001), and 2020–2024 (OR: 10.46; 95% CI: 2.39–45.87; *P* = 0.002).

**Table 2:** Multivariable logistic regression of ROSC, event survival and survival to hospital discharge in paediatric OHCA cases.

	**ROSC**		**Event survival**		**Survival to hospital discharge**	
	**Odds ratio (95% CI)**	***p*-value**	**Odds ratio (95% CI)**	***p*-value**	**Odds ratio (95% CI)**	***p*-value**
Age in years	1.05 (0.95–1.16)	0.348	1.02 (0.94–1.10)	0.667	1.01 (0.93–1.09)	0.876
**Sex**						
*Female*	Ref	Ref	Ref	Ref	Ref	Ref
*Male*	0.18 (0.05–0.65)	0.009	0.46 (0.17–1.25)	0.129	0.52 (0.20–1.38)	0.192
Public location	4.42 (1.16–16.87)	0.030	2.00 (0.66–6.03)	0.218	2.37 (0.80–7.03)	0.118
Metropolitan region	3.49 (1.06–11.49)	0.040	2.52 (0.94–6.71)	0.065	1.64 (0.61–4.43)	0.328
Bystander witnessed	3.26 (0.85–12.54)	0.085	3.31 (1.07–10.24)	0.037	3.76 (1.19–11.92)	0.024
Bystander CPR	1.30 (0.34–4.89)	0.701	1.56 (0.49–4.91)	0.450	1.91 (0.59–6.23)	0.284
Presumed cardiac aetiology	1.21 (0.30–4.79)	0.788	1.02 (0.32–3.18)	0.979	1.77 (0.55–5.64)	0.336
**First defibrillation by**						
*EMS*	Ref	Ref	Ref	Ref	Ref	Ref
*PAD*	0.71 (0.09–5.75)	0.746	0.69 (0.16–2.95)	0.620	1.21 (0.30–4.90)	0.786
**Year category**						
*2000–2004*	Ref	Ref	Ref	Ref	Ref	Ref
*2005–2009*	7.10 (1.71–29.58)	0.007	4.82 (1.39–16.75)	0.013	5.18 (1.35–19.96)	0.017
*2010–2014*	11.86 (1.75–80.53)	0.011	8.62 (1.69–43.88)	0.010	6.42 (1.26–32.76)	0.025
*2015–2019*	158.63 (21.37–1177.51)	<0.001	18.31 (4.61–72.73)	<0.001	10.79 (2.72–42.78)	0.001
*2020–2024*	80.62 (10.21–636.82)	<0.001	32.50 (6.29–167.99)	<0.001	10.46 (2.39–45.87)	0.002

Table 3 shows the results of the sensitivity analysis consisting of multivariable models with adjustment for the time to first shock. In these models, time to first shock was significantly associated with survival to hospital discharge (OR 0.92; 95% CI: 0.85–0.97; *P* = 0.020), event survival (OR 0.91; 95% CI: 0.85–0.98; *P* = 0.015) and prehospital ROSC (OR 0.88; 95% CI: 0.80–0.97; *P* = 0.007). In these models, first shocks by PAD were also not associated with any outcome. Other estimates remained similar to the primary model.

**Table 3:** Multivariable logistic regression of ROSC, event survival and survival to hospital discharge in paediatric OHCA cases, including call to first shock time.

	**ROSC**		**Event survival**		**Survival to hospital discharge**	
	**Odds ratio (95% CI)**	***p*-value**	**Odds ratio (95% CI)**	***p*-value**	**Odds ratio (95% CI)**	***p*-value**
Age in years	1.06 (0.96–1.18)	0.258	1.02 (0.94–1.11)	0.596	1.01 (0.93–1.10)	0.822
**Sex**						
*Female*	Ref	Ref	Ref	Ref	Ref	Ref
*Male*	0.18 (0.05–0.72)	0.015	0.49 (0.18–1.36)	0.171	0.52 (0.20–1.40)	0.199
Public location	4.41 (1.08–17.99)	0.039	1.83 (0.59–5.65)	0.294	2.08 (0.69–6.33)	0.196
Metropolitan region	2.25 (0.61–8.31)	0.223	1.87 (0.66–5.31)	0.243	1.31 (0.46–3.76)	0.609
Bystander witnessed	5.48 (1.24–24.27)	0.025	4.47 (1.36–14.64)	0.013	4.96 (1.50–16.47)	0.009
Bystander CPR	1.35 (0.34–5.41)	0.671	1.67 (0.51–5.48)	0.400	2.06 (0.60–7.02)	0.249
Presumed cardiac aetiology	1.17 (0.28–4.92)	0.835	0.99 (0.31–3.15)	0.980	1.66 (0.51–5.40)	0.401
**First defibrillation by**						
*EMS*	Ref	Ref	Ref	Ref	Ref	Ref
*PAD*	0.41 (0.04–4.11)	0.445	0.49 (0.11–2.22)	0.354	0.96 (0.22–4.10)	0.954
Call to first shock time (per min increase)	0.88 (0.80–0.97)	0.007	0.91 (0.85–0.98)	0.015	0.92 (0.85–0.97)	0.020
**Year category**						
*2000–2004*	Ref	Ref	Ref	Ref	Ref	Ref
*2005–2009*	6.14 (1.34–28.17)	0.019	4.20 (1.13–15.54)	0.032	5.66 (1.37–23.38)	0.017
*2010–2014*	15.56 (1.91–126.43)	0.010	9.56 (1.72–53.26)	0.010	8.07 (1.45–44.75)	0.017
*2015–2019*	191.12 (21.31–1713.98)	<0.001	17.24 (4.08–72.76)	<0.001	11.60 (2.76–48.60)	0.001
*2020–2024*	72.67 (8.25–640.39)	<0.001	29.62 (5.30–165.51)	<0.001	10.44 (2.26–48.15)	0.003

## Discussion

In this 25-year retrospective study of children experiencing OHCA, we found that while PAD was associated with improved unadjusted outcomes such as survival to hospital discharge, event survival and ROSC, these associations did not remain significant after adjustment for potential confounders. Instead, factors such as a bystander-witnessed arrest, time to first shock, and year of arrest were strongly associated with patient outcomes after paediatric OHCA from initial shockable rhythms. We observed higher unadjusted survival outcomes for patients shocked by PAD in this study indicating that improved outcomes were likely driven by temporal confounding and differences in baseline characteristics. Importantly, we observed a large temporal increase in the odds of survival, which was not explained, by increasing use of PAD. While PAD was not independently associated with improved outcomes in this cohort, our models suggest that reducing the time to first shock remains a key factor driving survival to hospital discharge outcomes in children.

A number of factors may help explain our results. First, improvements in both prehospital and in-hospital care over the past 25-years in the region may have contributed to better survival outcomes.^14^, ^17^, ^19^, ^20^ Second, while the increased use of PAD may have played a role in improving survival, our sample size may have been too small to confirm a statistically significant effect. Interestingly, in our adjusted models, first shocks by PAD were inversely associated with ROSC and event survival, although these estimates had wide confidence intervals. Third, while PAD use may have modest effects on survival, it’s possible that larger treatment effects may be observed on neurological outcomes, which were not collected in this study. Finally, the benefits associated with PAD may be influenced by the broader context in which it is used— the majority of patients who were exposed to initial shocks from PAD in our study arrested in public locations, were witnessed to arrest and almost all had bystander CPR.

There are similarities and differences between our findings and earlier studies of PAD use in children experiencing OHCA. Our results align with an earlier observational study from Japan of 128 initially shockable OHCA in school-aged children, which found that PAD shocks were not independently associated with favourable neurological survival at 1-month (AOR: 0.49; 95% CI: 0.12–2.02), 1-month survival (AOR: 0.59; 95% CI: 0.15–2.23), or ROSC (AOR: 0.61; 95% CI: 0.14–2.76) after adjustment for baseline differences.^7^ However, that study did identify that shorter time to shock was an important prognosticator of improved outcome, and patients with PAD shocks had markedly shorter shock intervals compared to EMS shocks (3.3 vs. 12.9 mins). Another retrospective observational study from the USA involving 3900 children aged <18 with non-traumatic OHCA, found that while bystander CPR was associated with improved survival, PAD application was not an independent predictor of survival in their multivariable analysis. This may be related to their included population, which consisted of a high number of infants who suffered cardiac arrests at home, were unwitnessed and had a non-shockable rhythm, which limits the potential impact of PAD on outcomes.^21^

In contrast, a cohort study of 971 children under 18 years with OHCA from the USA, found that PAD application was associated with significantly higher rates of survival to hospital discharge (adjusted 35.2% (95% CI: 25.8%–44.5%) and neurologically favourable survival (adjusted 31.1% (95% CI: 22.5%–39.7%), but this benefit was mostly seen in lower-risk (more affluent) neighbourhoods.^22^ The authors hypothesised that PAD application and improved outcomes were closely linked to factors like neighbourhood income, education, and overall access to emergency response resources.^22^ Similarly, in a Japanese study of 100 children aged 1–17 years with OHCA and an initial shockable rhythm found that patients treated with bystander CPR and PAD application (with and without shocks delivered) were associated with improved neurological survival at 1-month (AOR 3.17; 95% CI: 1.40–7.174, *p* = 0.005), 1-month survival (AOR 3.19; 95% CI: 1.40–7.24, *P* = 0.005), and ROSC (AOR 5.46; 95% CI: 2.32–12.87, *P* < 0.001) compared to children treated with bystander CPR alone.^5^ Additional studies from Japan also suggest a statistically significant association between PAD shocks and favourable neurological outcome.^23^, ^24^

Collectively, these findings suggest that PAD shocks may have a greater influence on functional recovery than on survival alone in paediatric OHCA. However, in Japan, previous research has focused mainly on school aged children, where almost all schools are equipped with at least one AED and require teachers and staff members to undertake basic life support training.^6^, ^23^, ^24^, ^25^ As a result, trained staff may be better trained to recognise OHCA and initiate CPR and defibrillation faster.^26^, ^27^

Notably, PAD shocks in our study were exclusively administered to children (30.4%) and adolescents (69.6%), with no cases involving infants. This likely reflects the fact that infants are more likely to arrest at home, where PAD access is limited and EMS remains the primary shock provider.^28^, ^29^ Therefore, the concentration of PAD use in public settings may further disadvantage individual outcomes for arrests occurring in private residences, where EMS reliance remains high and response times longer. To reduce the time between cardiac arrest and first shock, several strategies can be implemented. This includes utilising smartphone alerting technology to alert trained community volunteers to nearby cardiac arrests.^30^

The findings of this study highlight two important considerations. Firstly, our findings underscore the critical importance of minimising the time to first shock in children experiencing OHCA. Efforts to reduce the time interval from collapse to defibrillation—whether through PAD or by enhancing EMS response times—should remain a key priority for EMS systems and community-based interventions aiming to improve outcomes in this vulnerable population. Secondly, while PADs reduce time to defibrillation, our results suggest that this alone may not be sufficient to improve survival in paediatric OHCA. Future efforts may need to focus not just on increasing PAD availability, but on enhancing system-wide factors like CPR quality, and tailored emergency response protocols for children. Future studies should explore whether targeted interventions in private locations could increase the role of PAD in paediatric OHCA.

### Limitations

This study has several limitations that require consideration. First, the small sample size (*n* = 140) limited statistical power and resulted in wide confidence intervals in adjusted models. Second, temporal trends were restricted by the lack of PAD use in early years; less than five cases occurred between 2005 and 2009 and none before 2005. Third, the absence of neurological outcome data prevented evaluation of functional recovery, an important outcome in paediatric OHCA. Additionally, residual confounding may persist despite multivariable adjustments, as unmeasured factors (e.g., bystander training quality, AED maintenance, or in-hospital care variations) could influence outcomes. The underrepresentation of infants in PAD cases (0%) and the predominance of public location arrests may limit generalisability to private residences and younger age groups.

## Conclusion

PAD use in children experiencing OHCA has increased significantly over the past two decades, particularly in public settings. While PAD halved the time to first defibrillation in this study, PAD alone was not independently associated with improved survival. This may be due to the small number of PAD cases and the high rate of favourable arrest factors, such as witnessed arrests, public location, and bystander CPR. Our models indicate that the time to first defibrillation remains a strong predictor of survival in children, and this supports the ongoing use and expansion of PAD in children. Future studies with larger cohorts, detailed neurological assessments, and broader geographic representation are needed to validate these findings.

## Ethics

Ethics was approved by the Monash University Human Research Ethics Committee (Project ID 45498) and Ambulance Victoria’s Research Committee.

## CRediT authorship contribution statement

**Nora Abu-Obeid:** Writing – review & editing, Writing – original draft, Visualization, Project administration, Methodology, Investigation, Formal analysis, Data curation, Conceptualization. **Zainab Alqudah:** Writing – review & editing, Validation, Supervision, Methodology, Conceptualization. **Ashanti Dantanarayana:** Writing – review & editing, Validation, Resources, Data curation. **Ziad Nehme:** Writing – review & editing, Validation, Supervision, Resources, Methodology, Funding acquisition, Formal analysis, Data curation, Conceptualization.

## Funding

ZN is funded by National Health and Medical Research Council (NHMRC) and National Heart Foundation (NHF) Fellowships.

## Declaration of competing interest

None declared.
